# Gene Variability and Vulnerability to Alcoholism

**Published:** 1995

**Authors:** John J. Doria

**Affiliations:** John J. Doria, M.S., is a science editor for Alcohol Health & Research World

**Keywords:** gene, AOD use susceptibility, AOD dependence, metabolism, neurotransmitters, receptors, dopamine, serotonin, endogenous opioids

## Abstract

Variability within genes may explain why so many differences in alcoholism exist between individuals and among populations. Researchers studying this phenomenon have focused on genes that may affect both the way the body metabolizes alcohol and the way the brain responds to alcohol, thereby increasing or decreasing the likelihood of addiction. Gene variants may ultimately be used as markers of alcoholism vulnerability and as guides to help match patients to the most appropriate alcoholism therapies.

Evidence for the familial transmission of alcoholism has stimulated researchers to search extensively for specific genes that may confer vulnerability to alcoholism. Genetic analysis of behavioral disorders such as alcoholism is, however, especially difficult. Unlike single-gene disorders such as Huntington’s disease,^1^ behavioral traits appear to be influenced by the interaction of several genes, each of which may exert only a small effect (Plomin 1990). Social, cultural, and other nongenetic influences interact with genetic vulnerability to cause alcoholism (Devor 1993).

Despite the importance of nongenetic influences, however, the disease of alcoholism always occurs within a matrix of genetically determined mechanisms that differ between individuals and between populations. The basis for these differences is polymorphism, the existence of one or more variant forms (i.e., alleles) of the same gene. These inborn genetic differences serve as a starting point for understanding the web of interactions that lead to alcoholism (Goldman 1995).

Various methods exist for testing the contributions of individual genes to alcoholism vulnerability; these methods are described in other articles in this issue (see the Tools of Genetic Research section, beginning on page 190). Researchers have focused their attention on genes that may affect the way the body processes (i.e., metabolizes) alcohol and the way the brain responds to alcohol to increase or diminish the potential for addiction (Froehlich 1995).

## Genes for Alcohol Metabolism

Alcohol is metabolized in the liver by two enzymes acting sequentially. First, alcohol dehydrogenase (ADH) converts alcohol to a chemical called acetaldehyde. Acetaldehyde, in turn, is converted to acetate by aldehyde dehydrogenase (ALDH) (Goldman and Enoch 1990). Acetaldehyde is a toxic substance that may be partially responsible for some of the adverse medical consequences of alcohol consumption (Lieber 1994).

After consuming alcohol, certain Asian populations experience facial flushing, palpitations, headache, dizziness, and nausea. The flushing reaction is associated primarily with a defective variant of ALDH that allows the accumulation of acetaldehyde in the blood and tissues after alcohol consumption (Thomasson et al. 1993). In addition, researchers have detected an ADH variant that metabolizes alcohol at an increased rate, thereby contributing to acetaldehyde buildup. Although either enzyme variant can cause the alcohol flushing reaction, their effects are also additive (Thomasson et al. 1993). These effects are believed to deter drinking among those who experience them.^2^ Scientists have studied *ADH* and *ALDH* alleles extensively in various populations. (For a review, see the article by Wall and Ehlers, pp. 184–189.)

## Genes Related to Nerve Cell Communication

All brain activity results from communication between nerve cells. Alcoholism researchers have concentrated on the pathways of nerve cell communication most likely to assist in regulating alcohol consumption and promoting alcoholism. Among these are pathways that use the neurotransmitters^3^ dopamine and serotonin as well as the endogenous opioids.

### Dopamine

Dopamine is a neurotransmitter whose many functions include facilitating learning and motivation, processes that are central to developing addiction to alcohol and other drugs (AOD’s) (Di Chiara 1995).

#### Dopamine Metabolism

Dopamine released into a synapse binds rapidly to its receptors and is transported quickly back into the nerve cell that released it (i.e., reuptake). Following reuptake, some dopamine is stored for subsequent rerelease; the rest is metabolized by the enzyme monoamine oxidase (MAO). This enzyme occurs in two forms, or isoenzymes: MAO–A and MAO–B, which are encoded by two similar but distinct genes located close together on the same chromosome.

The precise roles of the two isoenzymes have not been fully clarified, although both MAO–A and MAO–B can metabolize dopamine. Lowered MAO–B activity has been detected frequently among alcoholics and their close relatives, compared with higher MAO–B activity among nonalcoholics. This difference could be attributed to polymorphism within the *MAO–B* gene. Such polymorphism may represent variation in the DNA sequence that codes for the enzyme’s structure or in the DNA sequence that regulates the gene’s activity (Devor et al. 1993). In either case, if variation in MAO–B activity can be related to specific DNA sequence variation within the *MAO–B* gene, the altered DNA sequence might serve as a genetic marker to help identify persons at risk for alcoholism (Devor et al. 1993).^4^

#### Dopamine Receptors

Five types of dopamine receptors have been identified in the brain and have been designated D_1_ through D_5_. Although all five receptors respond to dopamine, their specific properties and functions differ slightly from one another (Mansour et al. 1995). The dopamine receptor D_2_ plays a role in reinforcement, a key component of the process by which a behavior, such as alcohol consumption, becomes habitual (Di Chiara 1995). In 1990, Blum and colleagues reported a strong population association^5^ between alcoholism and a specific allele (A1) of the gene that directs the synthesis of D_2_ (the gene is known as *DRD_2_*). Specifically, researchers observed allele A1 of *DRD_2_* more frequently in a group of alcoholics compared with a group of nonalcoholic subjects (i.e., controls) (for a discussion of population association methods, see the article by Goate, pp. 217–220).

Subsequent research found this population association to be much weaker than originally reported (Parsian et al. 1991; Blum et al. 1991, 1993) or failed to find an association at all (Goldman et al. 1992, 1993; Gelernter 1993; Cook et al. 1992). In addition, Gejman and colleagues (1994) examined the DNA sequence of *DRD_2_* from large samples of alcoholics and schizophrenics and detected no variation in the coding segment of the gene that could account for an association with alcoholism.

Spurious results in population association studies may result from failure to match test subjects adequately to controls for ethnicity or other factors that may affect overall genetic composition. Significantly, in this regard, studies conducted in ethnically well-defined populations have generally failed to show an association between *DRD_2_* and alcoholism (Goldman et al. 1992; Arinami et al. 1993). According to Goldman and colleagues (1993), differences in *DRD_2_* polymorphism among diverse populations are sufficient to account for spurious population associations or, conversely, to obscure such associations that may exist.

Despite the failure to replicate the initial research on *DRD_2_*, the central role dopamine plays in the addiction process ensures that research on dopamine receptor genes will continue. Researchers have reported associations between *DRD_2_* and alcoholism-related phenomena (Comings et al. 1991), including drug abuse (Smith et al. 1992; Noble 1993) and aberrations of brain electrical activity (Noble et al. 1994). Variation also has been detected in the D_3_ (Rietschel et al. 1993) and D_4_ (Lichter et al. 1993) receptors, although the significance of this finding is unclear.

### Serotonin

Many investigators believe that alcoholism is related to the levels in the brain of the neurotransmitter serotonin (Litten and Allen 1991). Serotonin helps regulate such functions as bodily rhythms, food and water intake, sexual response, and aggression. Brain serotonin concentrations, which are difficult to measure directly, can be estimated by measuring the concentration in cerebrospinal fluid (CSF)^6^ of 5-hydroxyindoleacetic acid (5–HIAA), a product of serotonin metabolism. Reduced brain serotonin function, as indicated by low CSF 5–HIAA concentration, is associated with increased risk of violent behavior, suicide, depression, and alcoholism among people with psychiatric disorders as well as the general population (reviewed in Pihl and Peterson 1993). These manifestations appear to be causally related: The presence of one appears to increase the risk for any or all of the others (reviewed in Virkkunen and Linnoila 1993 and in Tollefson 1995). In addition, alcoholism and other problems involving impulsive behavior appear to be inherited together in families (Cloninger et al. 1985; Bohman 1978).

Family studies suggest that genetic polymorphism influences the function of serotonin and its receptors. Therefore, researchers are attempting to identify specific alleles that may influence vulnerability to alcoholism or behaviors that may be associated with alcoholism (Nielsen et al. 1994; Virkkunen et al. 1994*a*,*b*).

#### Genes for Serotonin Synthesis and Metabolism

Variations in serotonin levels and activity among different populations may be caused by polymorphism in genes that control serotonin synthesis and metabolism. Many of the genes potentially important in controlling serotonin metabolism have been cloned, including the gene that codes for the enzyme tryptophan hydroxylase (TPH) (Nielsen et al. 1994). TPH initiates the synthesis of serotonin from tryptophan, a chemical found in food. An allele of the TPH gene has been associated with low CSF 5–HIAA concentrations along with suicidal behavior among a group of alcoholic prisoners incarcerated in Finland for criminal offenses involving impulsivity (Nielsen et al. 1994). However, Abbar and colleagues (1995) did not find an association between TPH polymorphism and suicidal behaviors among patients in a French hospital.

MAO–A, discussed previously with respect to dopamine, also metabolizes serotonin. In a study of a single large family, researchers linked deficient enzymatic activity of MAO–A to a minor alteration in the DNA sequence of the *MAO–A* gene. Behavioral manifestations included borderline mental retardation, impulsive aggression, arson, attempted rape, and exhibitionism. This symptom cluster does not specifically include alcoholism and has not yet been reported in other populations (Brunner et al. 1993; Goldman 1995).

The genes for both MAO–A and MAO–B have been cloned (reviewed in Nielsen et al. 1994). However, both genes are on the female sex (i.e., X) chromosome. Because a high percentage of alcoholism is transferred from father to son, it is unlikely that genes located on the X chromosome account for more than a small fraction of the genetic vulnerability to alcoholism; nonetheless, *MAO–A* and *MAO–B* gene polymorphisms could be involved in the genetic liability of a subgroup of alcoholics (Goldman 1995).

Also cloned are the gene for aromatic L-amino acid decarboxylase (Sumi-Ichinose et al. 1992), another enzyme involved in serotonin synthesis, and the gene for the serotonin transporter, a protein in the nerve cell membrane involved in serotonin reuptake (Hoffman et al. 1991). Effects on alcoholism of possible polymorphisms at these genes have not been established.

#### Genes for Serotonin Receptors

Researchers have identified seven major classes of serotonin receptors, designated 5HT_1_ through 5HT_7_ (Grant 1995). These families have been further divided into subtypes, at least eight of which have been cloned (Goldman 1995). Receptor 5HT_3_ plays a role in the acute and chronic effects of AOD’s on the nervous system and also has been implicated in psychiatric illnesses such as schizophrenia, anxiety, and Alzheimer’s disease (Grant 1995). When activated by serotonin, 5HT_3_ functions to regulate communication along nerve cell pathways by influencing the release of certain neurotransmitters into synapses. Among the neurotransmitters regulated by 5HT_3_ is dopamine, discussed previously. Researchers have suggested that the direct action of alcohol on 5HT_3_ leads to increased dopamine activity in areas of the brain involved in reinforcement, potentially contributing to the development of addiction behaviors (Johnson and Cowen 1993; Grant 1995). Researchers are studying variations of other serotonin receptor genes for possible associations with alcoholism (Blanchard et al. 1992; Lappalainen et al. 1995).

### Genes Related to Endogenous Opioids

Endogenous opioids are neurotransmitters in the human nervous system that produce effects similar to those of morphine and other opiates. Evidence suggests that alcohol-induced activation of the endogenous opioid system may affect alcohol reinforcement and drinking behavior (Froehlich 1995). This theory is supported by the effectiveness of naltrexone, an opiate antagonist, in helping prevent relapse in patients being treated for alcoholism (Volpicelli et al. 1992). Animal studies suggest that a genetic predisposition toward alcohol consumption may be accompanied by increased responsiveness of the endogenous opioid system to alcohol (De Waele et al. 1992). In one study, alcohol consumption produced a significant increase in blood levels of beta-endorphin in subjects with significant family histories of alcoholism, but not in families without histories of alcoholism. The mechanism of this difference is unknown, although the results indicate that the basis of the difference is genetic (Gianoulakis et al. 1989).

## Conclusions

The causes and manifestations of alcoholism may differ significantly among individuals. Apart from environmental influences, multiple genes may each contribute to the disorder, and some researchers have suggested that certain families may have their own unique mixture of genes responsible for alcoholism vulnerability (Plomin 1990).

Population and molecular biology studies among humans will continue to shed light on the disorder. Animal studies will provide additional evidence, for example, by mapping quantitative trait loci (QTL), genes that together contribute to an effect although their individual effects may be minuscule. By studying QTLs in rodents, researchers may help identify genes that should be studied in humans. QTLs have been identified provisionally for alcohol withdrawal, alcohol preference, and alcohol sensitivity; evidence indicates that QTLs may be under common genetic control (Crabbe et al. 1994; also see the article by Grisel and Crabbe, pp. 220–227).

New directions for genetic analysis may be suggested by results of the Collaborative Study on the Genetics of Alcoholism (COGA). This comprehensive program, involving six research centers across the United States, is investigating the genetic contribution to alcoholism vulnerability. Final analysis of COGA data may reveal potential alcoholism-related genes unrelated to any genes previously thought relevant to alcoholism. (See the article on COGA, pp. 228–236.)

Future study of genetic variants also might focus on some of the more specific and directly heritable alcoholism-related traits, such as variants of brain electrical activity (Enoch et al. in press). Ultimately, gene variants may be used as markers of alcoholism vulnerability and as guides to help match patients to appropriate alcoholism therapies (Goldman 1995).

## Footnotes

1 For a definition of this and other technical terms used in this article, see central glossary, pp. 182–183.

2 Cultural factors also contribute to differences in drinking patterns between populations.

3 Communicating nerve cells generally are separated by a microscopic gap called a synapse. Nerve cells release molecules called neurotransmitters into the synapse, where they bind to receptor proteins in the walls of other nerve cells and thereby carry messages from cell to cell.

4 Alternate explanations for differences in MAO–B activity have been postulated, some of which do not require genetic involvement.

5 A population association indicates that a particular allele occurs more frequently in a group of subjects who have the disease, compared with a group of control subjects, who do not have the disease.

6 Cerebrospinal fluid surrounds the brain and spinal cord and fills the cavities within the brain.
